# Supplementary figures and images for: Metabolic and pathologic profiles of human LSS deficiency recapitulated in mice
Source: PLoS Genet. 2020 Feb 26;16(2):e1008628. doi: 10.1371/journal.pgen.1008628 (PMC7062289; doi:10.1371/journal.pgen.1008628)

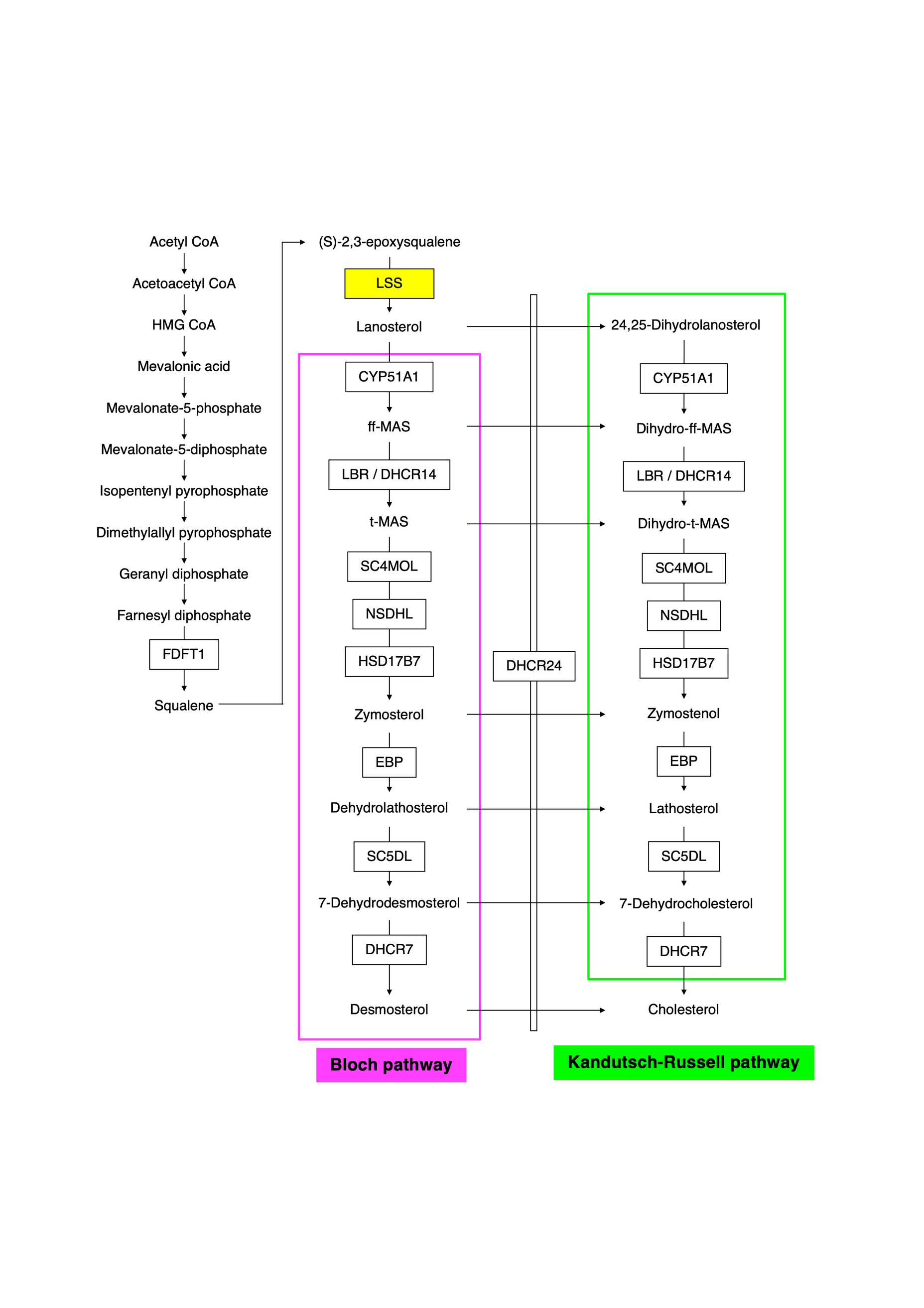

Supplement: S1 Fig — Cholesterol is synthesized from acetyl CoA via the Bloch or Kandutsch-Russell pathway. In this synthesis pathway, LSS converts (S)-2,3-epoxysqualene to lanosterol. The abbreviations are as follows: FDFT1, farnesyldiphosphate farnesyltransferase 1; ff-MAS, follicular fluid meiosis-activating sterol; t-MAS, testis-meiosis activating sterol; LBR, lamin B receptor; SC4MOL, sterol C4-methyloxidase-like; NSDHL, NAD(P)H steroid dehydrogenase-like protein; HSD17B7, 17-beta-hydroxysteroid dehydrogenase; EBP, emopamil-binding protein; SC5DL, sterol C5-desaturase; DHCR7, 7-dehydrocholesterol reductase; and DHCR24, 24-dehydrocholesterol reductase. (TIF) [file pgen.1008628.s001.tif]

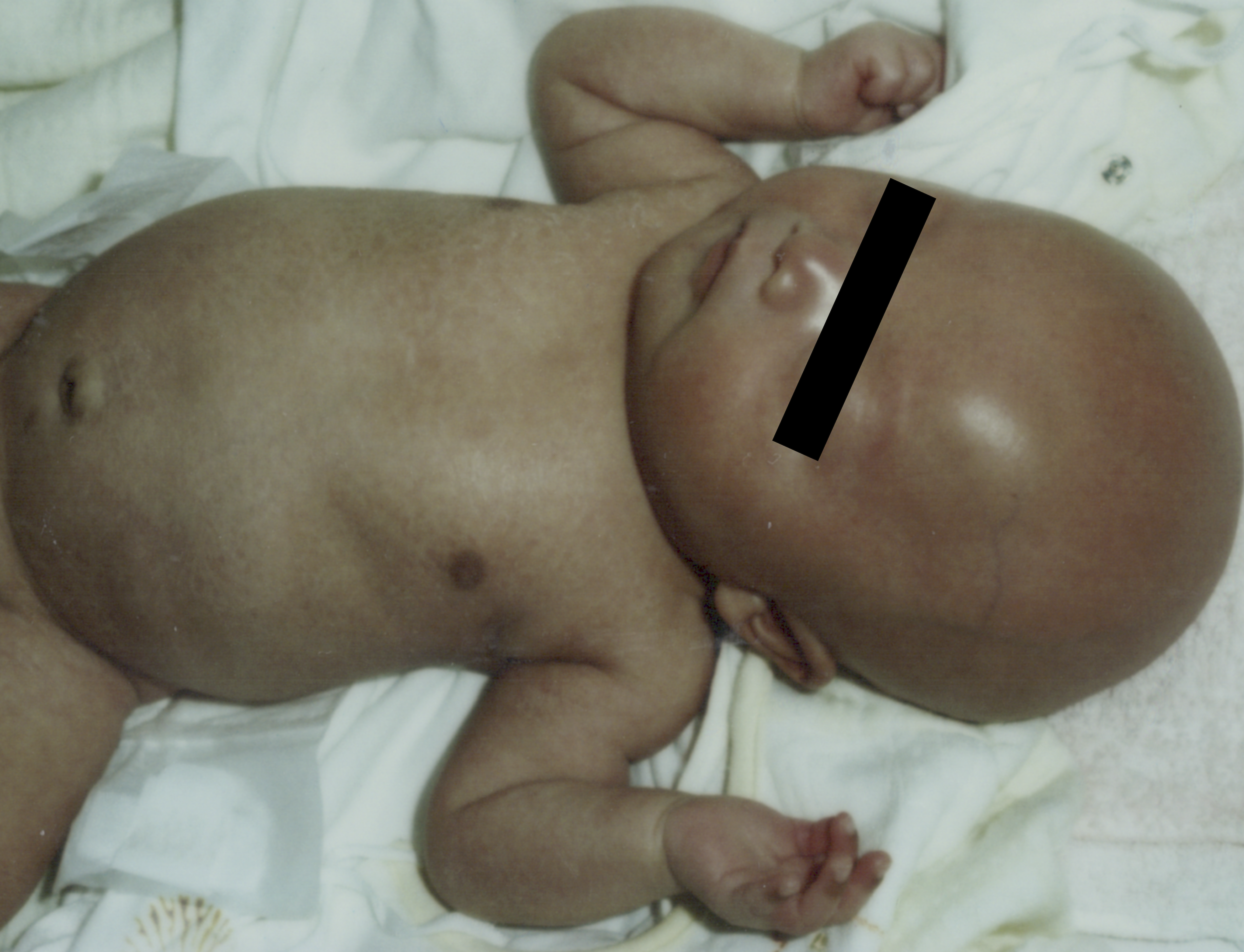

Supplement: S2 Fig — The patient exhibited ichthyosis-like scales on the skin of his chest, abdomen and upper extremities but not on his face and head. (TIF) [file pgen.1008628.s002.tif]

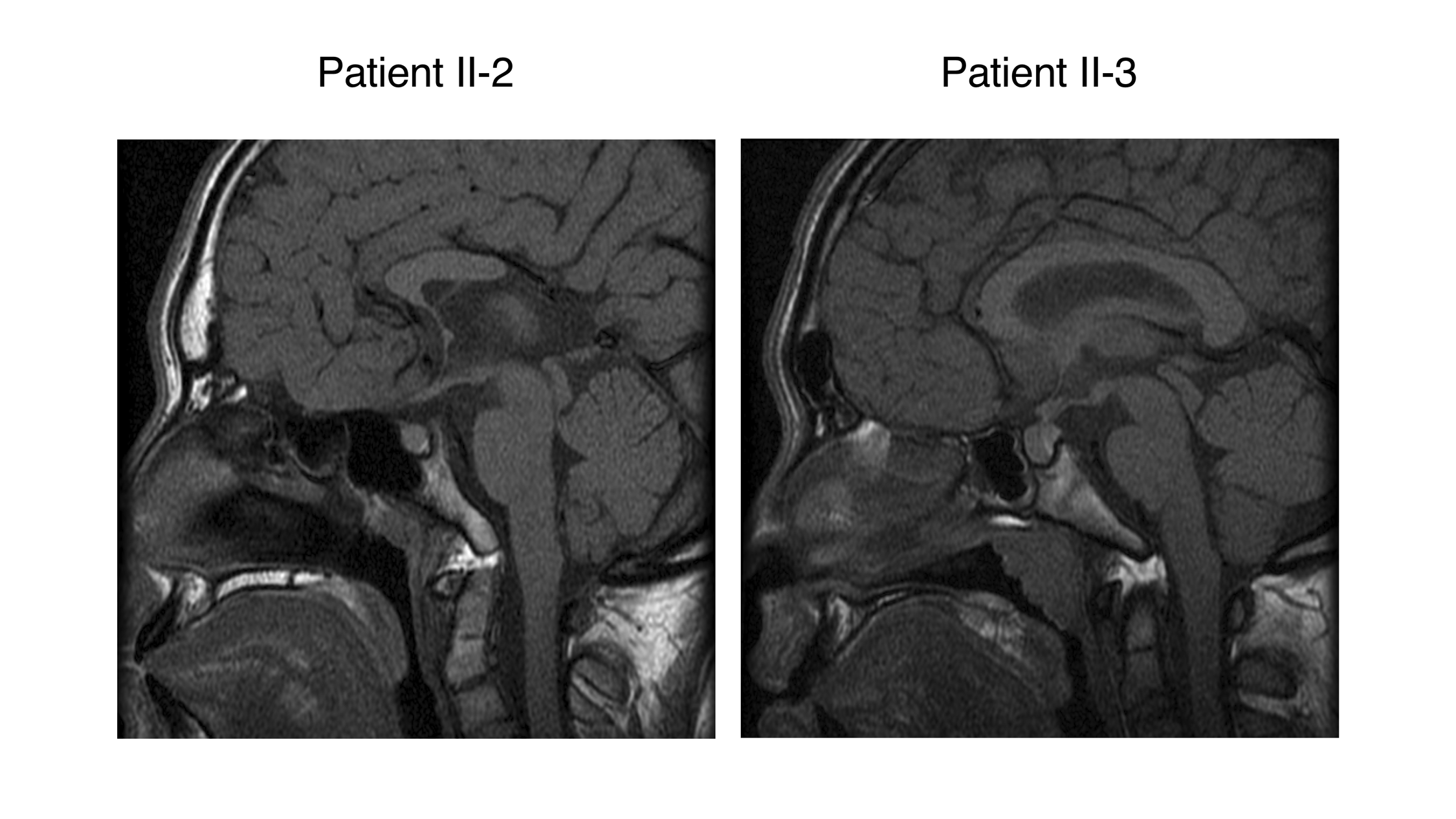

Supplement: S3 Fig — Sagittal T1-weighted head MRI shows partial agenesis of the corpus callosum (from the posterior midbody to splenium) in patient II-2, while the MRI shows a normal corpus callosum in patient II-3. (TIF) [file pgen.1008628.s003.tif]

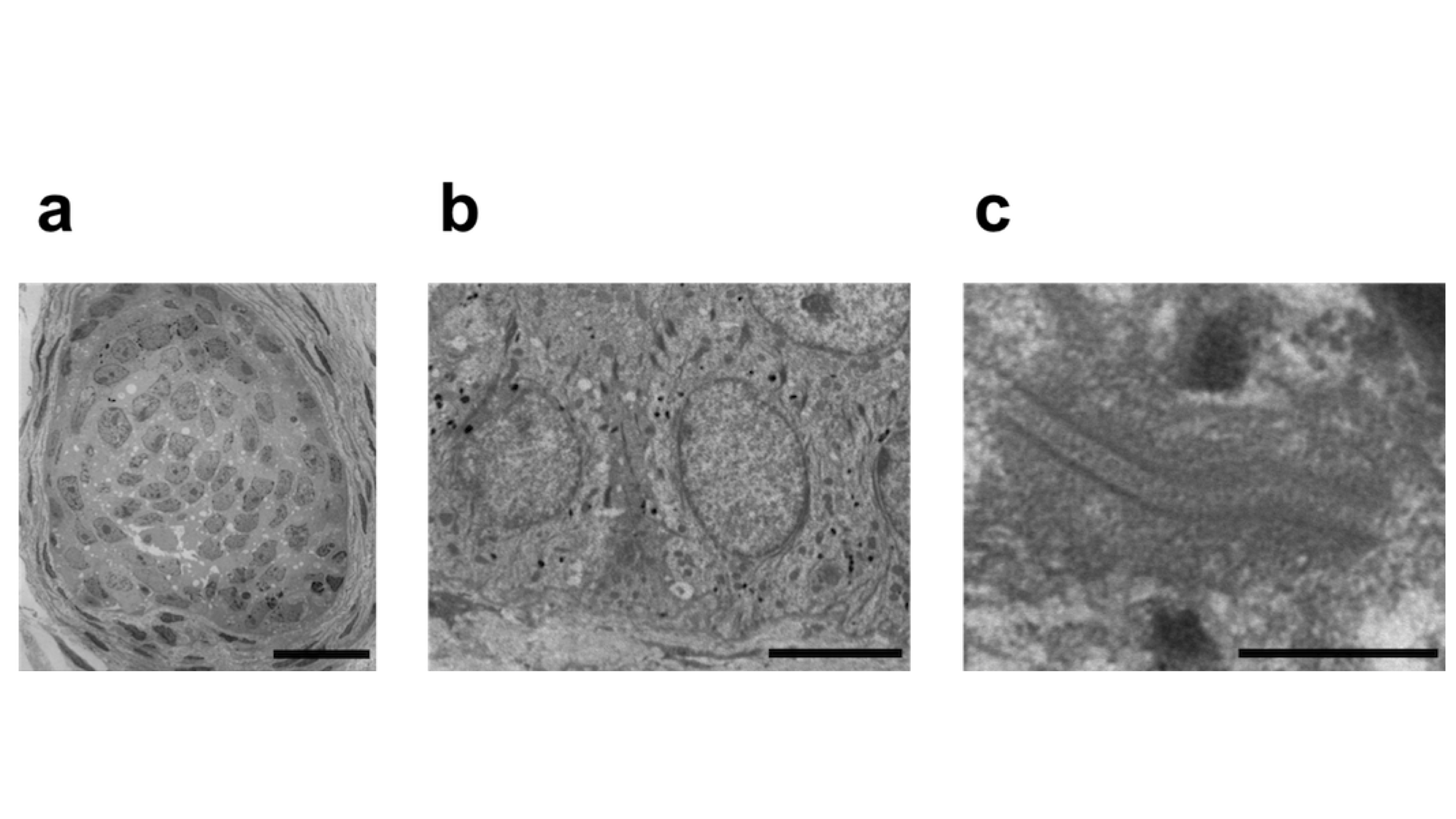

Supplement: S4 Fig — (a) Hair matrix of the patient. The number of melanin granules was small. The inner root sheath was thin. (b) The basement layer cells were tightly adhered to each other. (c) The desmosome was well developed. Scale bars = 200 μm (a), 50 μm (b), and 2.5 μm (c). (TIF) [file pgen.1008628.s004.tif]

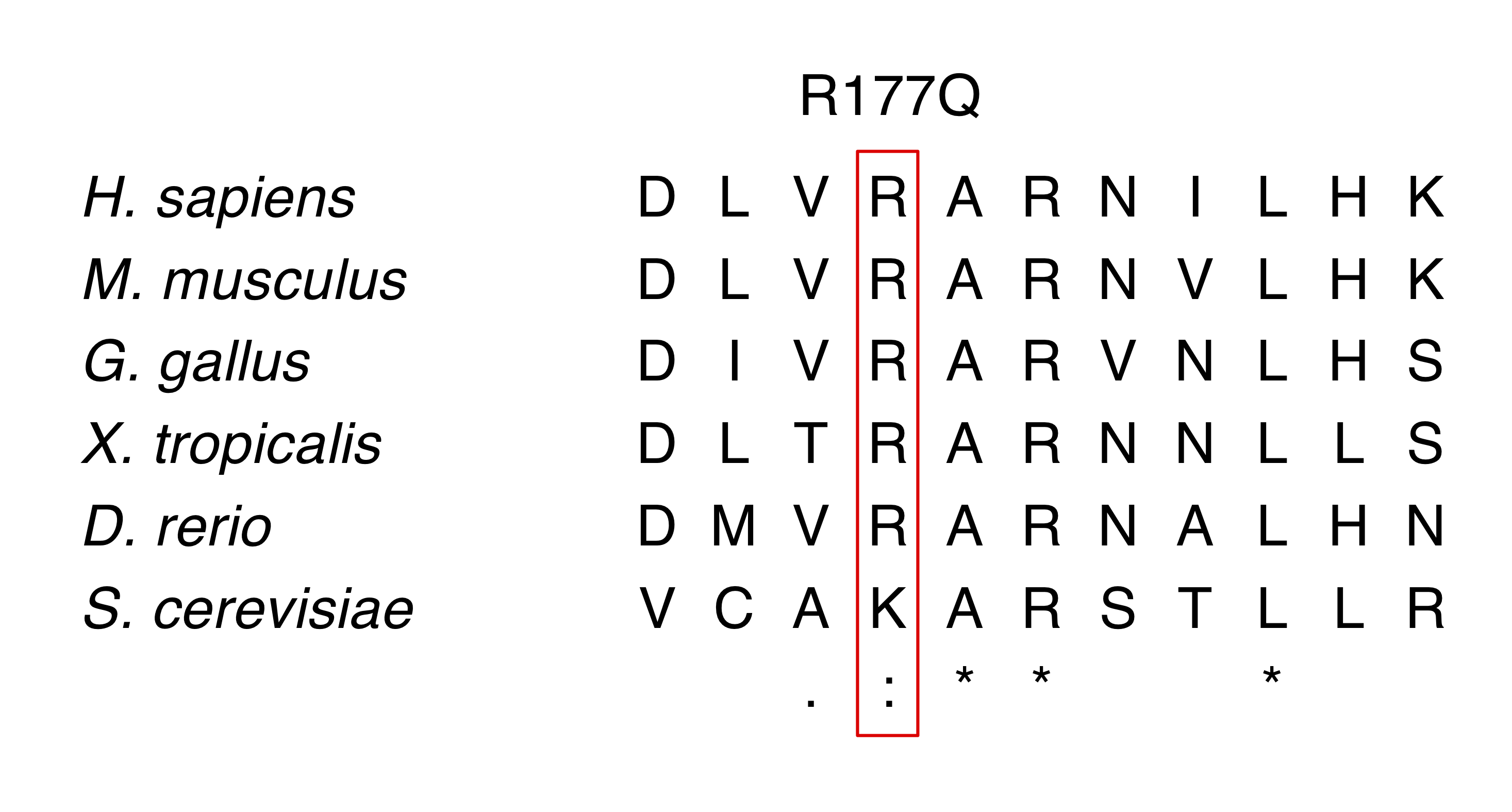

Supplement: S5 Fig — The R177Q residue is conserved in organisms from humans to zebrafish. LSS homologs were aligned with Clustal Omega. (TIF) [file pgen.1008628.s005.tif]

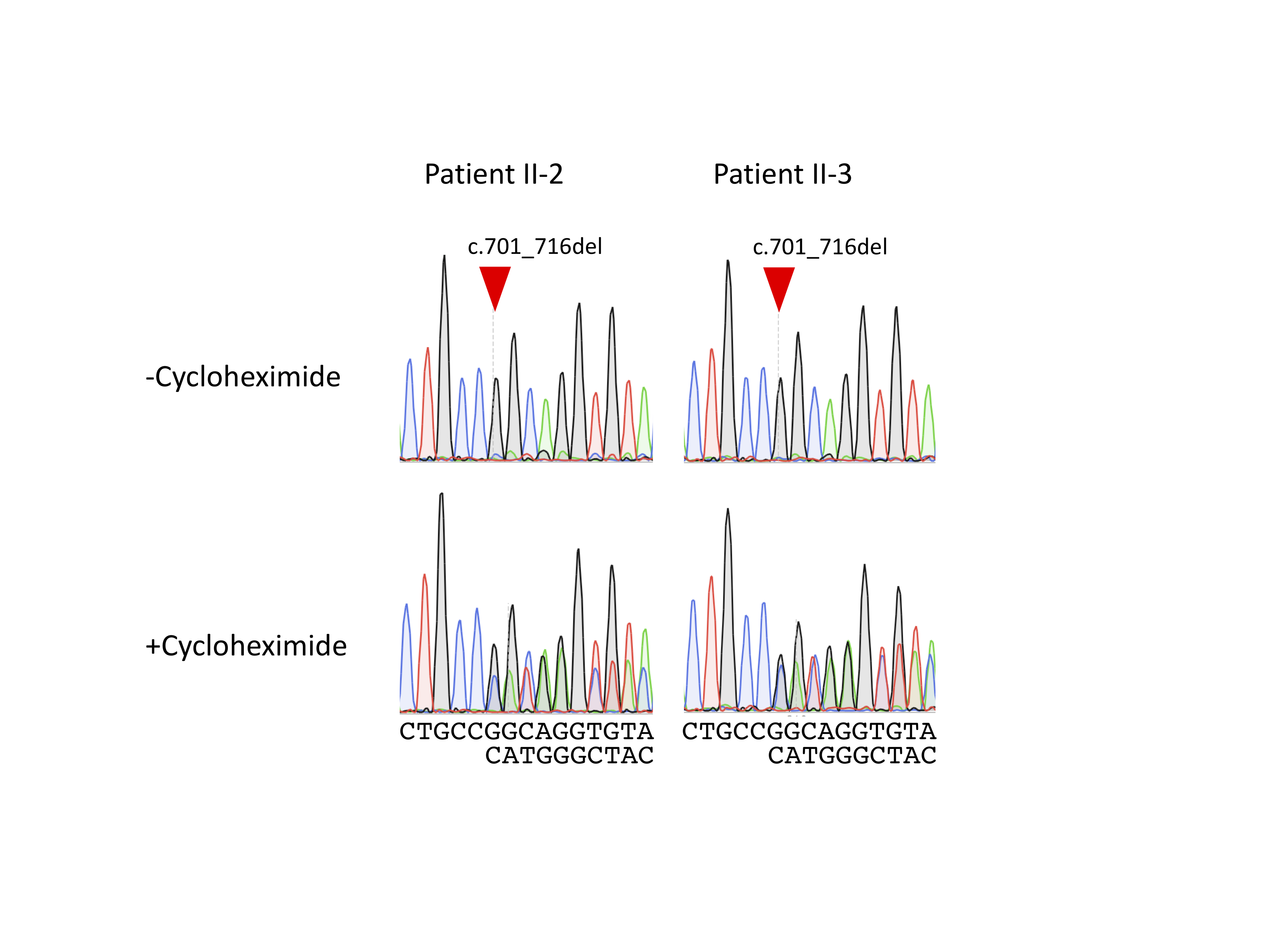

Supplement: S6 Fig — EBV-LCLs from patient II-2 and II-3 were treated with 100 μg/mL cycloheximide (037–20991, FUJIFILM Wako Pure Chemical Corporation, Osaka, Japan) for 4 hours to suppress NMD. Total RNA was extracted with an RNeasy Kit (Qiagen Inc., Valencia, CA, USA). RT-PCR was performed using a PrimeScript™ II High Fidelity RT-PCR Kit (TaKaRa, Shiga, Japan) to amplify the cDNA surrounding the c.701_716del mutation, which was followed by Sanger sequencing. (TIF) [file pgen.1008628.s006.tif]

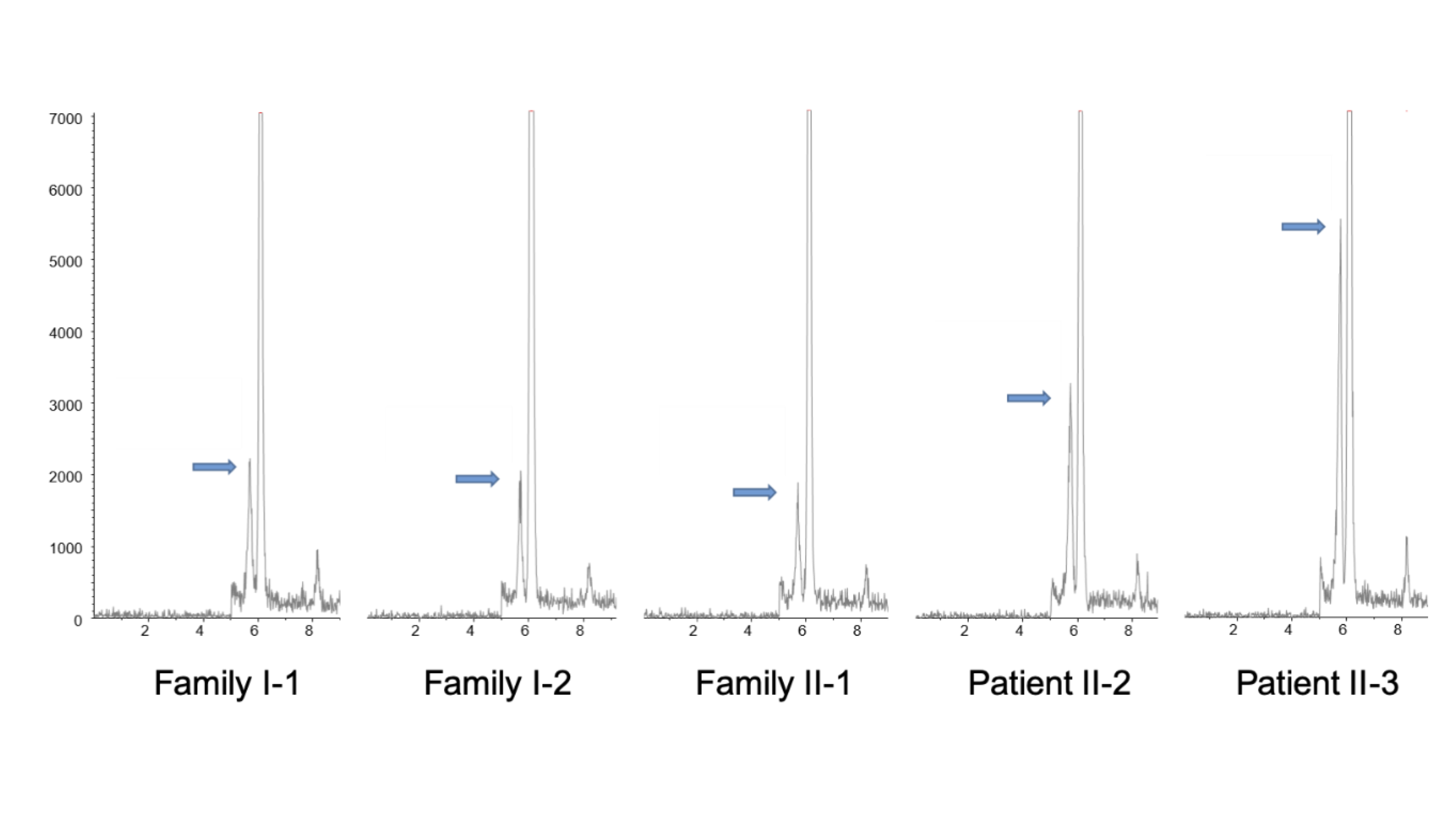

Supplement: S7 Fig — Blue arrows indicate peaks of 2,3-oxidosqualene. Lanosterol could not be detected by this analysis. (TIF) [file pgen.1008628.s007.tif]

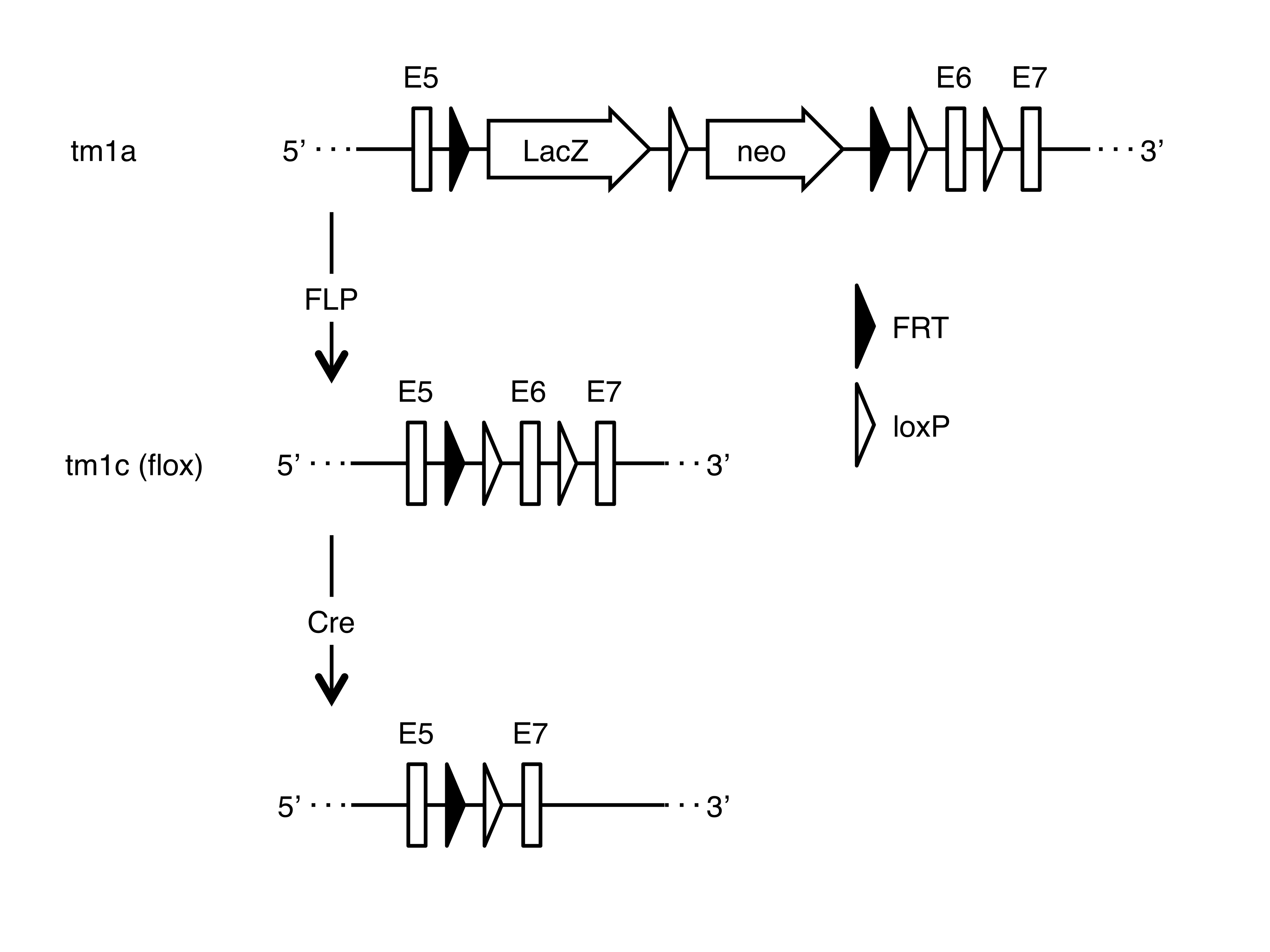

Supplement: S8 Fig — The tm1a allele was a knockout first allele, and it was converted to a conditional allele, tm1c (flox), by FLP-mediated recombination. The tm1c (flox) allele was converted to a deletion allele by Cre-mediated recombination. (TIF) [file pgen.1008628.s008.tif]

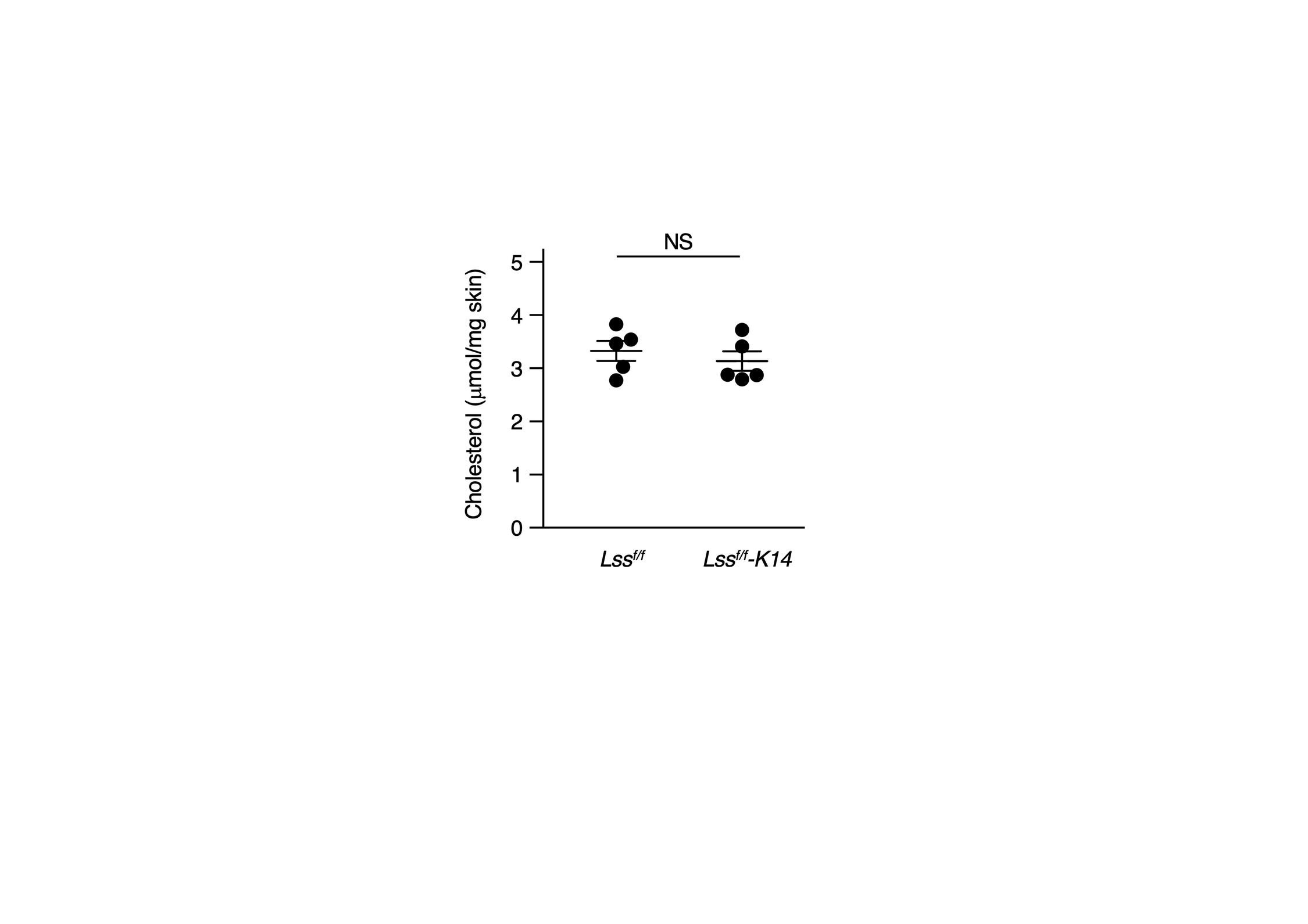

Supplement: S9 Fig — Mouse skin was gravimetrically weighed, and homogenates were prepared using 0.9% KCl containing 1 mM ethylenediaminetetraacetic acid. Total lipids were extracted from the homogenates using the Folch method [34, 35], and the extract was dissolved in 100 μL of 2-propanol. Human sebum was collected using acetone-soaked cotton pads [32], and it was dissolved in 200 μL of 2-propanol. The total cholesterol content in each sample was measured enzymatically with a commercial kit (Cholesterol E; Wako Pure Chemical Industries, Ltd., Osaka, Japan) according to the manufacturer’s protocol. Each bar represents the mean ± SEM (n = 5 per group). P-values were calculated using Student’s t-tests. NS, not significant. (TIF) [file pgen.1008628.s009.tif]

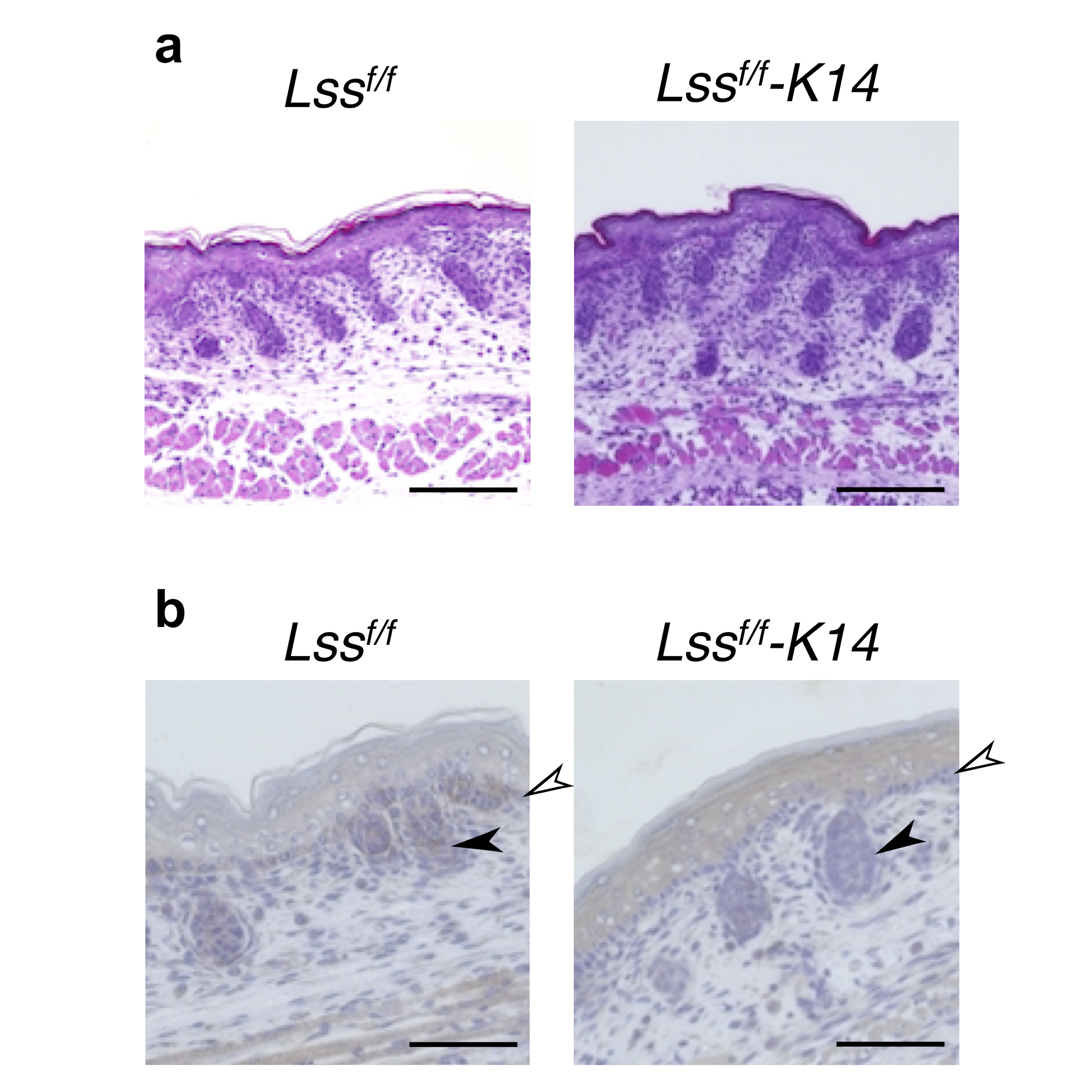

Supplement: S10 Fig — (a) Histological analysis did not show macroscopic differences in epidermal structure between the Lssf/f-K14 and Lssf/f mice. (b) Immunohistochemistry showed that expression of the LSS protein was decreased in the epidermal basement membrane (open arrowhead) and hair follicles (closed arrowhead) of the Lssf/f-K14 mice compared to the Lssf/f littermates. Scale bars = 100 μm. (TIF) [file pgen.1008628.s010.tif]

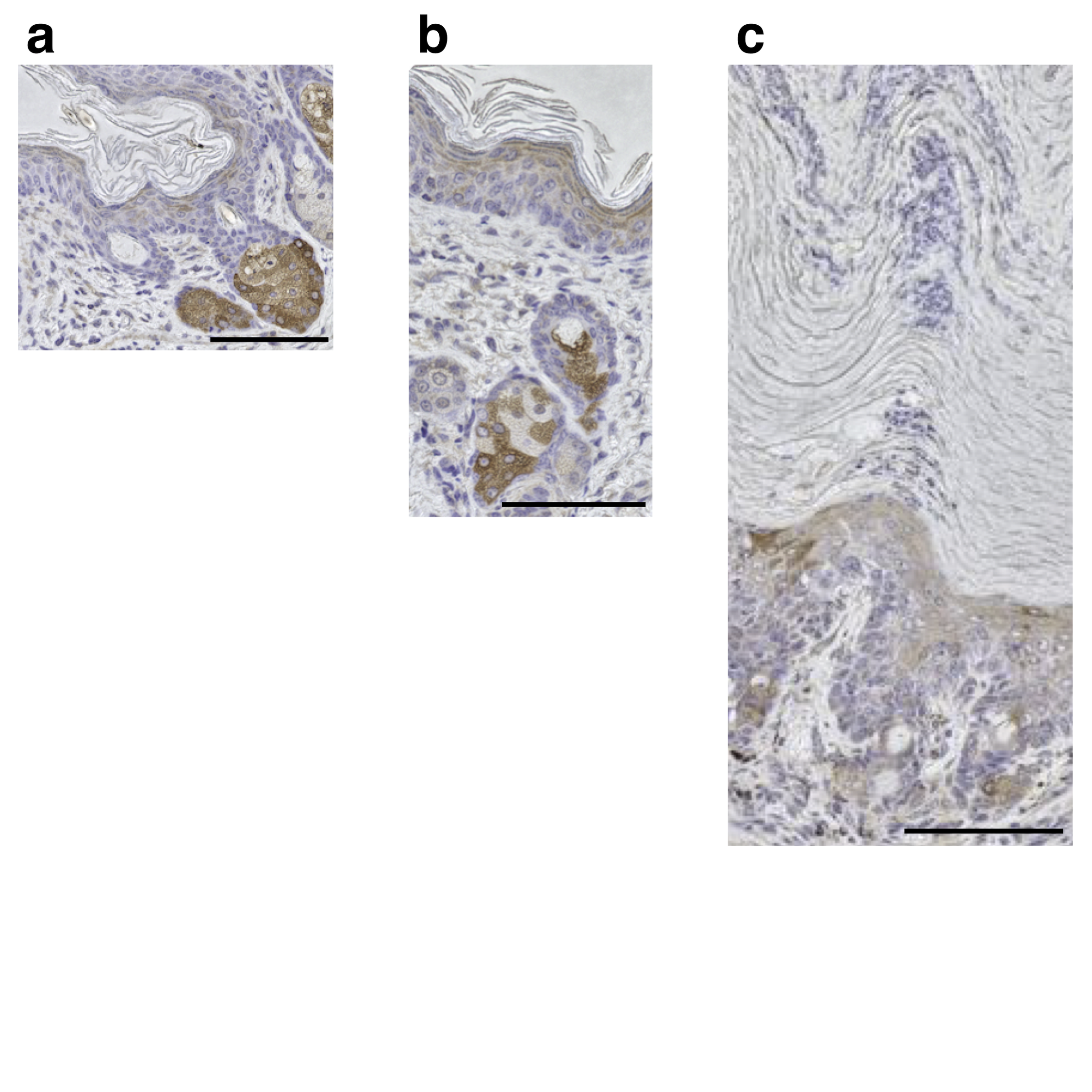

Supplement: S11 Fig — Keratosis is mild in epidermal areas near immunostained sebaceous cells (a, b). In contrast, severe keratosis is found in epidermal areas lacking immunostained sebaceous cells (c). Scale bars = 100 μm. (TIF) [file pgen.1008628.s011.tif]

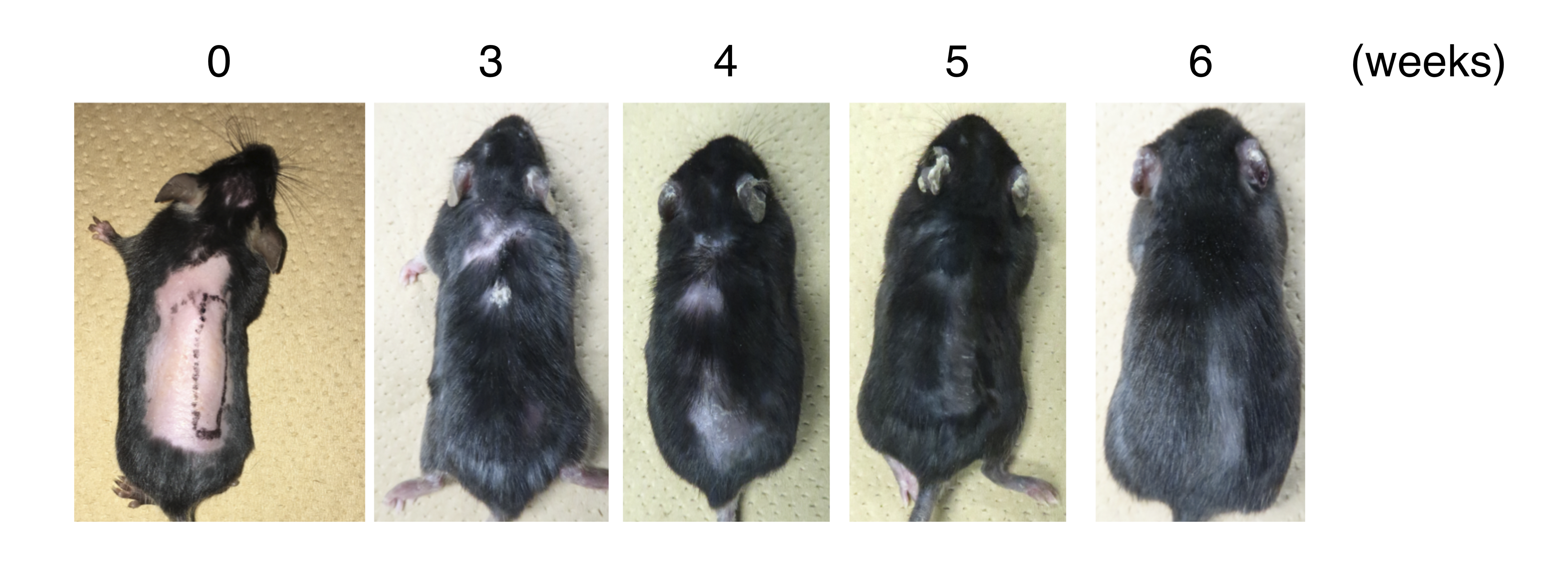

Supplement: S12 Fig — Images of Lssf/f-K14ERT mice arranged chronologically after topical application of 4-hydroxytamoxifen, which was applied within the black rectangle. Depilation gradually occurred after 3 weeks of 4-hydroxytamoxifen treatment. The depilation area was greatest after 4 weeks. Similar to the effects of intraperitoneal injection of tamoxifen, the hair gradually regrew. Gray hairs were mixed among the hairs in the recovered area. (TIF) [file pgen.1008628.s012.tif]

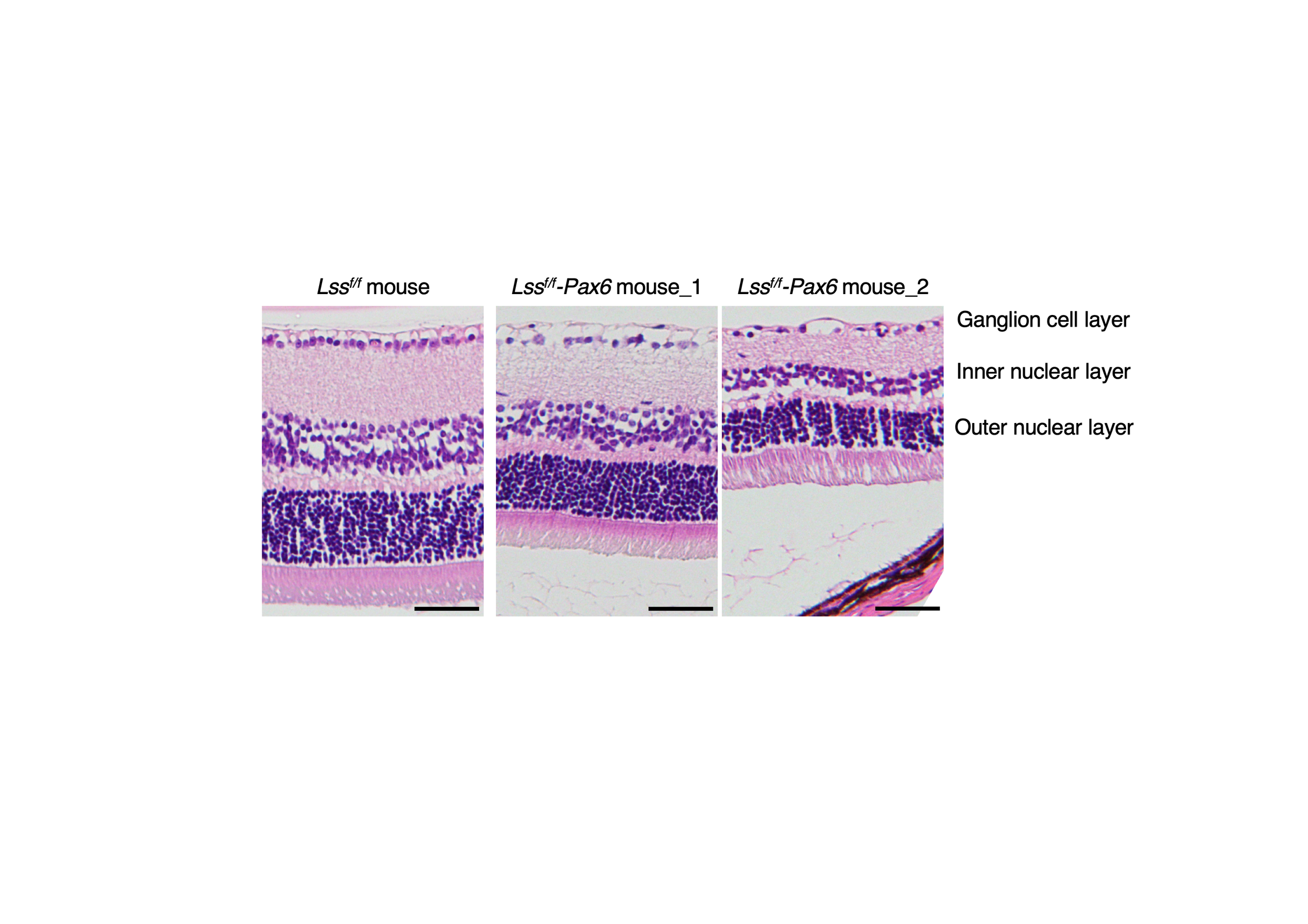

Supplement: S13 Fig — The inner retina, which is the inner nuclear layer and ganglion cell layer, was thin in Lssf/f-Pax6 mice compared to Lssf/f mice. There were fewer retinal ganglion cells in Lssf/f-Pax6 mice than there were in Lssf/f mice. In contrast, there was no obvious difference in the thickness of the outer nuclear layers between Lssf/f-Pax6 and Lssf/f mice. These pictures were captured using an Olympus BX53 microscope (Olympus, Tokyo, Japan). A 10x objective lens was used. Scale bars = 50 μm. (TIF) [file pgen.1008628.s013.tif]

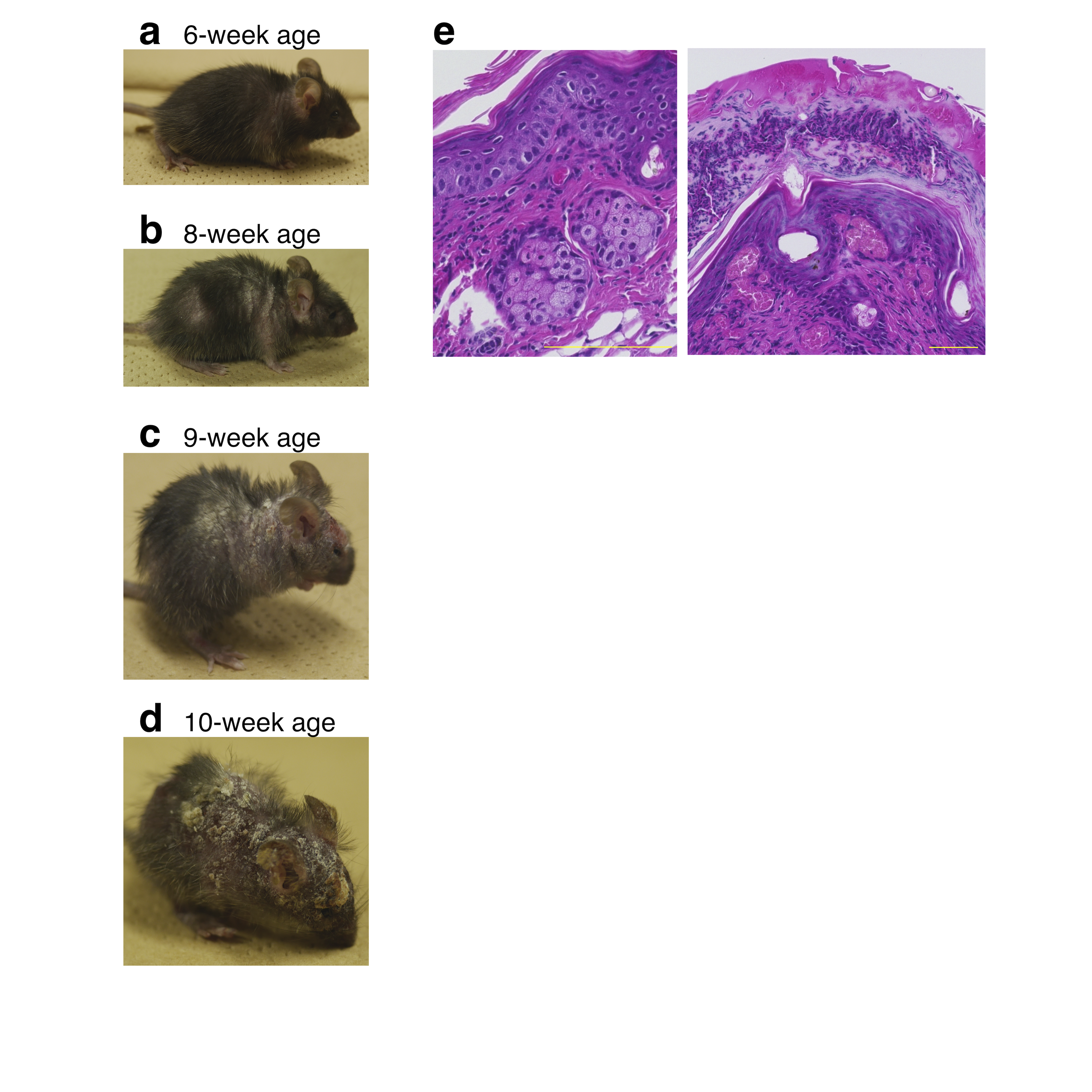

Supplement: S14 Fig — Sequential images of an adult Lssf/f-K14 mouse (a-d). At weaning, the mouse could not be differentiated from the other littermates. The mouse gradually lost hair after 6 weeks of age (a). Desquamation started at 9 weeks of age (c). A partial skin defect on the head with oozing was observed. The mouse died due to unknown causes at 10 weeks of age (d). Analysis of histological specimens from the dead mouse revealed severe hyperkeratosis, dyskeratosis, and hypertrophic sebaceous cells (e). Scale bars = 100 μm. (TIF) [file pgen.1008628.s014.tif]

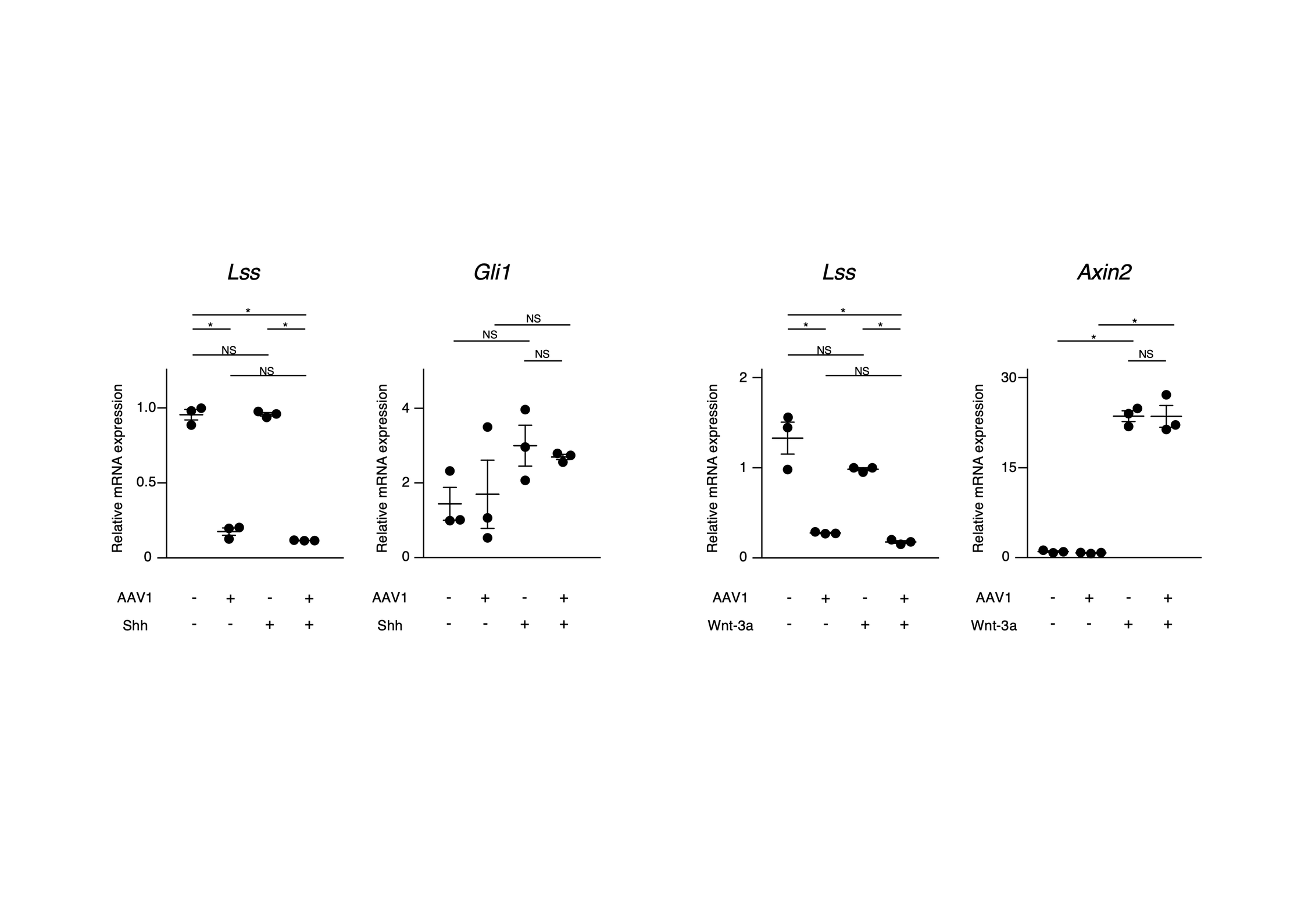

Supplement: S15 Fig — We measured the transcriptional responses of MEFs against the exogenous Shh or Wnt protein as previously described [27, 38] with minor modifications. Embryos (E13.5–14.5) were harvested by aseptic techniques from pregnant Lssf/f mice that had been mated with Lssf/f male mice. The heads, extremities, and tails were cut off, and the livers were removed. The remaining body parts were minced by scissors and transferred to a 50 mL tube with PBS. The tube was centrifuged at 200 g for 5 min. The supernatant was aspirated, and the pellet was resuspended with 1 mL 0.25% trypsin-EDTA per embryo. The suspension was incubated for 15 min in a 37°C water bath with shaking. Fetal bovine serum was added (the same volume as trypsin) to the suspension. After letting the suspension sit for several minutes and allowing the embryo fragments sank to the bottom of the tube, the serous part was removed first and filtered, and then the remaining thick part was passed through a 100 μm filter. The filtered solution was centrifuged at 200 g for 5 min. The supernatant was aspirated, the pellet was resuspended in DMEM, and the suspension was transferred to a dish. MEFs were plated at 5 × 10^4^ cells in 250 μL of DMEM per 48-well plate and transduced with AAV1 or a mock control (105548-AAV1 or 105537-AAV1, respectively, Addgene, Cambridge, MA, USA) at a multiplicity of infection of 5 ×10^5^. The next day, 250 μL of DMEM was added to each well. The following day, 2 μg/mL Shh (464-SH-025/CF, R&D systems, Minneapolis, MN, USA) or 100 ng/mL Wnt‐3a (1324-WN, R&D systems, Minneapolis, MN, USA) were added to the medium. Three days after transduction, total RNA was extracted with RNeasy (QIAGEN, Hilden, Germany), and reverse transcription was performed with a PrimeScript RT Reagent Kit with gDNA Eraser (TakaraBio, Shiga, Japan). cDNA was amplified with TaKaRa Ex Taq (RR001A, TaKaRa Bio Inc., Otsu, Japan) and PrimeTime qPCR probe assays (Integrated DNA Technologies, Coralville, IA, USA). qPCR primers and prob [file pgen.1008628.s015.tif]
