# Supplementary material for: Metabolic and pathologic profiles of human LSS deficiency recapitulated in mice
Source: PLoS Genet. 2020 Feb 26;16(2):e1008628. doi: 10.1371/journal.pgen.1008628 (PMC7062289; doi:10.1371/journal.pgen.1008628)
Supplement: S5 Table — (DOCX) [file pgen.1008628.s020.docx]

**S5 Table.**

**MS parameters used for LC-MS/MS analysis**

| Parameters | Lanosterol | (S)-2,3-Epoxysqualene |
| --- | --- | --- |
| Q1 mass (Da) | 411.0 | 427.2 |
| Q3 mass (Da) | 95.0 | 69.0 |
| Collision energy (V) | 49.0 | 62.0 |
| Collision cell exit potential (V) | 15.0 | 10.7 |
| Declustering potential (V) | 70.0 | |
| Entrance potential (V) | 10.0 | |
| Curtain gas (psi) | 30.0 | |
| Collision gas (psi) | 4 | |
| Ion spray voltage (V) | 5500.0 | |
| Temperature (℃) | 400.0 | |
| Ion source gas 1 (psi) | 40.0 | |
| Ion source gas 2 (psi) | 0.0 | |
