# Supplementary material for: Metabolic and pathologic profiles of human LSS deficiency recapitulated in mice
Source: PLoS Genet. 2020 Feb 26;16(2):e1008628. doi: 10.1371/journal.pgen.1008628 (PMC7062289; doi:10.1371/journal.pgen.1008628)
Supplement: S6 Table — The forward primers of Lss tm1a, Lss tm1c, and Lss tm1d were the same. The existence of the Pax6-Cre allele was confirmed using generic Cre primers and a probe. (DOCX) [file pgen.1008628.s021.docx]

**S6 Table.**

**Primers and probes**

| Target | Name | Sequence 5’ - 3’ |
| --- | --- | --- |
| *Lss wild* | Forward | GCTGCATGTTAGACTGTCCATGA |
|  | Reverse | GGAGCCTTGAGAAAGAAGGAG |
|  | Probe | 56-FAM/ATAAGCAGC/ZEN/ATCTTGGGCTCGGAT/3IABkFQ |
| *Lss tm1a* | Forward | GCGCATAACGATACCACGATATC |
|  | Reverse | CAGTGTTTGGGGCAAGTGTG |
|  | Probe | 56-FAM/AACTTCGAA/ZEN/CCCTTTCCCACACCA/3IABkFQ |
| *Lss tm1c* | Reverse | GTAGGCGGCATGAGTCACTTG |
|  | Probe | 56-FAM/ATGTCGAGA/ZEN/TATCTAGACCCAGCT/3IABkFQ |
| *Lss tm1d* | Reverse | GGTAAATGTTTGGGAAGTCCTAGAT |
|  | Probe | 56-FAM/ACAGGGATG/ZEN/GGATGAACTGATGGC/3IABkFQ |
| *K14-Cre*  *K14-CreERT* | Forward | GGAGATAGGATGCGTCAGGC |
|  | Reverse | CCATCAGCCCCTCGGCTC |
|  | Probe | 56-FAM/TCACATTCC/ZEN/TCTCAACATGCCTGCC/3IABkFQ |
| *Pax6-Cre* | Forward | GCGGTCTGGCAGTAAAAACTATC |
|  | Reverse | GTGAAACAGCATTGCTGTCACTT |
|  | Probe | 56-FAM/AAACATGCT/ZEN/TCATCGTCGGTCCGG/3IABkFQ |

The forward primers of *Lss tm1a*, *Lss tm1c*, and *Lss tm1d* were the same.

The existence of the *Pax6-Cre* allele was confirmed using generic Cre primers and a probe.
