# Supplementary material for: Metabolic and pathologic profiles of human LSS deficiency recapitulated in mice
Source: PLoS Genet. 2020 Feb 26;16(2):e1008628. doi: 10.1371/journal.pgen.1008628 (PMC7062289; doi:10.1371/journal.pgen.1008628)
Supplement: Attachment — Submitted filename: Response_to_Reviewers_LSS_PG_20191125_ak_NPGedited.docx [file pgen.1008628.s022.docx]

Point-to-point answers

**EDITORS**

*All three reviewers were enthusiastic about the major findings of this manuscript. However, a number of issues were identified that will require revision and/or clarification. These issues will need to be addressed in a point-by-point rebuttal. Lastly, the following issues in particular may require additional experimental data:*

**Response:** Thank you for your constructive comments and summarizing the points to address.

*- Photomicrographs of the hair and ocular structures in addition to the lens*

**Response:** We have supplemented photographs of the skin and retina in Figure 1 and S13 Figure. The inner retina was thin, and there were fewer retinal ganglion cells in *Lss^f/f^-Pax6* mice than there were in *Lss^f/f^* mice.

*- Measurements of sterol intermediates*

**Response:** We have added assays used to measure cholesterol and squalene levels in human sebum and mouse skin. These results have been included in S9 Figure and have been added throughout the manuscript. The dynamics of sterol should be key to elucidating the pathogenicity of LSS deficiency, but the comprehensive analysis of sterol intermediates of human sebum and mouse skin was technically difficult in this study. Further studies are needed to clarify the sterol profile in patients and mice.

*- Gene and or protein expression data exploring which signaling pathways, such as Wnt or Hh-related pathways, are impacted by LSS deficiency*

**Response:** We conducted additional experiments to determine the relationship between *Lss* knockout and Wnt/Shh signaling disturbance in MEFs from *Lss^f/f^* mice. AAV-Cre vectors successfully deleted the *Lss* gene and decreased *Lss* mRNA expression, but we could not find a transcriptional change in Wnt/Shh signaling (S15 Figure). Spatial or sequential analysis of the mouse skin might be required to provide evidence of abnormal Wnt/Shh signaling.

*- Inclusion of the suggested controls for the transfection experiments in hela cells*

**Response:** We agree that the overexpression studies do not always reflect the protein stability of the mutants under physiological conditions and that the knock-in/knock-out strategy is an ideal approach. We checked the levels of overexpressed FLAG-tagged LSS protein and intrinsic LSS protein (from the HeLa cells transfected with the “Mock vector”) not with an anti-FLAG antibody but with an anti-LSS antibody, unlike in Figure 1h. We observed a much higher level of the overexpressed wild-type LSS protein compared to intrinsic LSS protein levels (see a figure below). Therefore, we assume that the intrinsic LSS protein is negligible when testing the stability of mutant proteins as a first step. We can at least conclude that with the marked reduction of LSS expression in Epstein-Barr virus-transformed lymphoblastoid cell lines (EBV-LCLs) from affected patients, it seems that both of the patients’ alleles affect the stability of the LSS protein.

**Figure.** The levels of LSS proteins in the HeLa cells between wild-type LSS (FLAG-tagged) construct and mock (intrinsic LSS protein); Western blotting was performed not with an anti-FLAG antibody but with an anti-LSS antibody, unlike in Figure 1h.

*- Methods used for lanosterol and 2,3-epoxysqualene quantification*

**Response:** We have rechecked the conditions for the analysis and reworded them to establish a clearer focus. The details are included in the response to comment 4 of Reviewer 2.

**REVIEWER #1**

*The authors submit an interesting study whereby they describe two new patients with novel biallelic mutations in lanosterol synthetase (LSS) who exhibit hair and midline structural abnormalities and a concomitant increase in the substrate:product (S-2,3-epoxysqualene:lanosterol) ratio secondary to an enzymatic block. Additionally, the authors generate tissue-specific LSS-deficient mouse lines with hair/skin and lens abnormalities; the skin from these tissue-specific LSS-deficient mice demonstrate increased S-2,3-epoxysqualene:lanosterol ratios which may alter normal skin homeostasis and hair follicle cycling. The authors provide extensive data throughout the manuscript; however, there are some concerns:*

**Response:** We wish to express our appreciation for the reviewer’s insightful comments, which have helped us significantly improve our manuscript.

***Comment 1.*** *In Figure 1g, the authors illustrate the S-2,3-epoxysqualene:lanosterol ratio from the sebum of the affected patients, their unaffected family members, and controls. Given that cholesterol is essential in the formation of an intact skin barrier, it would be beneficial to show the values of all sterol intermediates and cholesterol in the sebum in these groups since one can speculate that these skin and hair abnormalities may be due to either a deficiency of cholesterol, a buildup of toxic intermediates, or both.*

**Response:** We succeeded in detecting cholesterol in the human sebum, while the cholesterol levels were not determined due to the cholesterol levels being so low. We have also added data on squalene/lanosterol in the sebum from the affected patients, their unaffected family members, and controls in Figure 1j. The added data indicated a disruption of LSS metabolism in the patients; while the squalene to lanosterol ratio does not enable differentiation of the carrier and the patient, the (S)-2,3-epoxysqualene to lanosterol ratio does. The measurements of other sterol intermediates are fascinating. However, in this study, we failed to detect/measure the intermediates, except for (S)-2,3-epoxysqualene, lanosterol, and squalene, partly because of the limited amount of sample. We are interested in analyzing the values of sterol intermediates in future studies.

***Comment 2.*** *In addition to the high magnification photomicrograph in Figure 1e, a lower magnification photomicrograph of the hair follicles is needed to assess the extent of hair follicle pathology.*

**Response:** We have added a lower magnification photomicrograph of the hair follicles in Figure 1e.

***Comment 3.*** *As stated above in Concern 1, values for the remainder of the sterol intermediates and cholesterol would be useful in explaining the pathophysiology seen in the LSSf/f-K14 mice illustrated in Figure 2.*

**Response:** We have also performed experiments to investigate cholesterol or sterol intermediate levels of skin in *Lss^f/f^-K14* and *Lss^f/f^* mice. The cholesterol levels were not significantly different between the *Lss^f/f^-K14* and the *Lss^f/f^* mice. We failed to detect/measure the intermediates, except for (S)-2,3-epoxysqualene, lanosterol, and squalene, as described in comment 1. Squalene is generally not detected in mouse skin, as shown in a previous report [PMID 5924301], and the same results were obtained in *Lss^f/f^-K14* and *Lss^f/f^* mice. We have added these results to the revised manuscript (page 10, line 191-193) and S9 Figure.

***Comment 4.*** *Have the authors considered treating the 4-week-old tamoxifen-treated LSSf/f-K14ERT mice that show hair loss and skin abnormalities with statins and/or cholesterol + statins? As the authors suggest in the Discussion, this therapy may reduce these intermediates and reverse the pathology seen in these mice, particularly since the hair follicle cycles continuously throughout the majority of a mammal’s lifetime.*

**Response:** Yes, we did consider this. We topically applied lanosterol or statin or supplied animals with customized chow supplemented with lanosterol or statin to prevent the skin lesions caused by the *Lss* knockout. These preliminary attempts were not successful (data not shown). Further experiments are planned to evaluate other reagents, combinations of reagents, reagent forms, treatment protocols and timing of treatment.

***Comment 5.*** *Figure 4 depicts the lens pathology seen in mice with LSS deficiency restricted to the lens. From the photomicrographs shown in Figure 4b, there appears to be pathology within other ocular/intraocular structures, such as within the cornea and retina, than in just the lens. Given Pax6-Cre is expressed during early eye development, loss of LSS within other ocular/intraocular tissues may not be surprising. It would be interesting to determine if the LSSf/f-Pax6 mice have retinal or other ocular/intraocular abnormalities besides those of the lens. Lastly, the authors do not state the age of these mice and do not show the LC-MS/MS data of lens or other ocular structures.*

**Response:** Accordingly, we have again reviewed ocular pathological abnormalities (except the lens) with ophthalmologists. The inner retina was thin in *Lss^f/f^-Pax6* mice compared to *Lss^f/f^* mice. We have added these findings to page 12, line 244-246 and S13 Figure.

Next, we have tried to measure lanosterol or sterol intermediate levels in mouse lenses, but we were unable, despite using the lipid extraction method for rat lenses [PMID 16440058]. There is, as far as we know, no report to date where sterol intermediates from mouse lens were measured. This is partly because the mouse lens might be too small to detect sterol intermediates. This information is included in page 12, line 246-248*.*

The age of these mice has been added in the legend of Figure 4.

***Comment 6.*** *Additional points include:*

*-Line 135: Is the variant supposed to be p.Arg177Gln instead of p.Arg177Glu?*

**Response:** We have changed “p.Arg177Glu” to “p.Arg177Gln”.

*-Line 226: Should it be “increased ratio of substrate to product” instead of decreased?*

**Response:** We have changed “decreased” to “increased”.

*-The flow of the overall manuscript is rather choppy at times and could use proofreading by a native English speaker for better flow and readability.*

**Response:** Prior to the first submission, our manuscript had been edited for English language usage, grammar, spelling and punctuation by one or more native English-speaking editors at Nature Research Editing Service. In this revised submission, the entire manuscript has been edited again by editors at Nature Research Editing Service. In the discussion section, we have moved the contents of the third paragraph of the previous manuscript into the first paragraph for better flow.

*Overall, the manuscript is interesting and not only provides more clarification of the tissue-specific pathophysiology seen in these malformation syndromes but also hints at diagnostic assays/therapeutic interventions for LSS deficiency*.

**Response:** Again, we are grateful for all of your insightful comments.

**REVIEWER #2**

*In the manuscript entitled "Metabolic and pathologic profiles of human LSS deficiency recapitulated in mice ", the authors demonstrate disruption of lanosterol synthetase in an inducible mouse model of LSS deficiency results in biochemical deficits and tissue specific changes present within affected human subjects. The work presented within the manuscript is important to the fields of cholesterol biosynthesis defects, rare disease research, and epidermal function. While the authors nicely demonstrate the identification of defined mutations within a small patient cohort of LSS deficiency and recapitulate some of the tissue deficits within a newly derived mouse model, the data presented are not likely of broad enough scientific significance for acceptance in PLOS Genetics. A significant advancement forward for LSS deficiency, such as identification of causative pathways downstream of LSS mutation or amelioration of pathological findings, would greatly enhance the significance of the manuscript.*

**Response:** We appreciate the reviewer’s insightful comments.

***Comment 1.*** *While the methodology utilized within the manuscript is solid, the data presented, including generation of an inducible Lss mouse model, is largely descriptive. While the inducible Lss mouse is an exciting development for elucidating the pathological mechanisms subsequent to Lss disruption, the addition of mechanistic data is really needed. Are there specific signaling pathways subsequent to Lss deletion that are pathogenic?*

**Response:** As requested, we performed additional experiments to unveil the pathological mechanism of LSS deficiency, which are shown in S15 Figure. We assessed Shh or Wnt signaling in MEFs derived from *Lss^f/f^* mice, and we found no significant differences between *Lss* knockout and wild-type MEFs in Shh or Wnt transcriptional activities (S15 Figure). These results might indicate that the underlying mechanism is not simply caused by the upregulation or downregulation of these pathways because spatial and gradient SHH or WNT signaling seems to be key in skin formation [PMID: 26771489, 27498049]. We have now revised the discussion section (page 15, line 315-321). We would like to address the involvement of Shh or Wnt using histological analysis in future studies.

***Comment 2.*** *It’s not clear the significance of the experiments detailing FLAG-tagged LSS mutations and changes in protein expression of specific mutants, particularly in HeLa cells that are demonstrated to already express LSS protein. How a transient transfection of a plasmid containing a mutant cDNA for LSS leads to decreased expression of native LSS is confusing. Further, why specific mutations affect this versus others is similarly confusing. Controls for this experiment for expression levels of the plasmids in an LSS null cell line would be critical.*

**Response:** We agree that the overexpression studies do not always reflect the protein stability of the mutants under physiological conditions and that a knock-in strategy is an ideal approach. We determined the levels of overexpressed FLAG-tagged LSS protein and intrinsic LSS protein (from the HeLa cells transfected with the “Mock vector”), and we used an anti-LSS antibody, not an anti-FLAG antibody, unlike in Figure 1h. We observed an exceptionally high level of wild-type LSS protein compared to that of intrinsic LSS protein (see a figure in the response for the editor). Therefore, we assume that the intrinsic LSS protein is negligible when testing the stability of mutant proteins as a first step. We can at least conclude that with the marked reduction of LSS expression in EBV-LCLs from affected patients, it seems that both of the patients’ alleles affect the stability of the LSS protein. Now, we have added the following text to the results (page 9, line 163-165):

“The result of *in vitro* LSS expression suggested that the mutant protein stability was affected, although caution must be taken because overexpression studies sometimes do not reflect the protein stability of the mutants under physiological conditions.”

We also agree that the overexpression analysis of other previously reported mutants is confusing. Accordingly, we have now removed the data (Figure 1h) and the description (page 9) of previously reported mutants.

***Comment 3.*** *The clinical findings from the two subjects identified are similar but not identical to the K14 mouse model used. The presence of cleft palate, midline anomalies, and corpus collosum agenesis in particular would not be affected by K14-Cre mediated deletion of Lss. Are these deficits present in the newly created Lss mutant mouse if a constitutive or embryonic Cre-driver used?*

**Response:** This is an interesting perspective. We have checked the cleft palate in *Lss^f/f^-K14* neonatal mice and *Lss^f/f^* littermates. The morbidity of the cleft palate was not significantly different between these mice. Although we focused on two core phenotypes, namely, cataracts and hypotrichosis in this study, reproducing midline anomalies or brain anomalies by new *Lss* conditional knock-out mice mediated by other embryonic Cre-drivers would provide elucidation of the pathogenicity of LSS deficiency. Analysis of these mice need to be addressed in the future.

***Comment 4.*** *The quantitation and detection of sterols is not clearly defined. I believe there are typos within the methods, including what temperature was utilized for compound ionization. Further, how the compounds of interest were identified (i.e. through comparison to a known standard?) was performed. The number of samples analyzed as biological vs. technical replicates is also not clear. Further, the explanation regarding detection of lanosterol and 2,3-epoxysqualene in plasma vs. sebum of patients is unclear. It’s not clear why lanosterol would be detected.*

**Response:** Thank you for your suggestion that the quantitation and detection of sterols are not clearly defined. We rechecked the parameters for MS analysis and confirmed that the parameters described in S5 Table were correct. The identification of compounds, as the reviewer suggested, was performed through comparison of peaks between the samples and commercial standards. We added a description about this in the revised manuscript as follows (page 18, line 391-394).

“Compounds were detected with atmospheric-pressure chemical ionization in positive ion mode and were identified through comparison of peaks between the samples and commercial standards. Compounds were quantified with external calibration curves using the corresponding standards. Further MS conditions are described in S5 Table”.

With regard to biological and technical replicates, we have conducted multiple technical replicates (i.e., multiple injections of the same standards) and have confirmed that such variations are minimal. For biological replicates, we have not conducted biological replicates based on the exact same biological samples (e.g., human sebum from the same individual) because the amount of biological samples analyzed in this study was limited. Alternatively, we analyzed multiple samples of similar biological origin, and as the results suggest, variations were determined to be rather small (Figure 1i, Figure 1j, Figure 2c, Figure 3c). With this in mind, we had not described the number of samples analyzed.

The discrepancy of detection of lanosterol and (S)-2,3-epoxysqualene in plasma vs. sebum of the patients is an interesting result. A possible explanation is that the activity of the downstream enzyme of LSS would differ by tissue. Lanosterol in the sebum might be metabolized more slowly than it is in the blood.

One speculation for why lanosterol could be detected in the sebum of the patients is that the p.Arg177Gln might be a hypomorphic variant that leads to mild production of lanosterol. We would like to establish an assay for measuring LSS enzyme activity to test these hypotheses.

***Comment 5.*** *For the early lethality of the Lss mouse presumably associated with dehydration, it would be of interest to know if lethality of the mouse could be prevented by administration of fluids. Further, if this is the presumed cause of death and the identified patients clearly have epidermal loss of Lss, it’s unclear why their lifespans are extended in comparison to the mouse.*

**Response:** It is an intriguing idea to avoid neonatal lethality of *Lss^f/f^-K14* mice by administration of fluids. Adult *Lss^f/f^-K14* mice would be a good model to research pathogenicity or development therapies for patients with LSS deficiency. We are preparing the rearing cages, which enables maintaining high humidity, although this attempt has been unsuccessful so far. We agree that the discrepancy in lifetime between the patients and the *Lss* knockout mice is unexplained. It is possible that different residual LSS enzyme activities in the epidermis cause different epidermal phenotypes. The LSS enzyme activity of our patients might be enough to form a skin barrier (but are insufficient to develop hair follicles), while almost total depletion of LSS might cause skin barrier dysfunction, as in *Lss^f/f^-K14* mice. The creation of *Lss* hypomorphic mice or the accumulation of patients with an LSS deficiency would clarify this disparity.

**REVIEWER #3**

*This manuscript by Wada et al. is a carefully crafted insight into LSS deficiency. The authors identified two sibling patients with hypotrichosis and other abnormalities, and performed exome sequencing leading to the identification of LSS mutations as the likely cause. Consistent with this, they found an increase in the substrate of LSS ((S)-2,2-epoxysqualene) in sebum from the patients – previous efforts had not been able to confirm a lack of LSS activity. They then made skin and lens-specific Lss-deficient mice to show that LSS deficiency results in similar skin problems and cataracts, as indicated in humans. This work is the first to confirm that a lack of LSS activity in the affected tissues leads to the specific phenotypes observed in patients with LSS deficiency. This is significant and has important implications for the treatment of this disease, as well as broader implications for other cholesterol synthesis enzyme defects associated with diseases. I have only minor comments, which I list below.*

**Response:** Thank you for your detailed comments and constructive suggestions.

***Minor comments:***

***Comment 1.*** *Page 5, line 64: please rephrase “Cholesterol is an essential nutrient” as this is not strictly correct.*

**Response:** We have replaced this sentence with “Cholesterol is essential for the regulation of biological functions in animals”.

***Comment 2.*** *Supp Fig 1: Only LBR is listed – please also include DHCR14 (these enzymes catalyse the same step). Zymosterol is listed twice – it should be zymostenol in the Kandutsch-Russell pathway. Dehydrodesmosterol should be 7-dehydrodesmosterol. Dihydrolanosterol is, I think, more commonly referred to as 24,25-dihydrolanosterol.*

**Response:** We have corrected S1 Figure according to your advice.

***Comment 3.*** *Page 5, line 73-74: The sentence about the 24 patients is confusing – suggest rephrasing to “Out of 24 patients, all had either cataracts (5/24) or hypotrichosis (20/24), and one had both”.*

**Response:** Accordingly, we have replaced the sentence with your suggestion.

***Comment 4.*** *Page 5, line 86: Should this be a citation to the publication by Dickinson et al 2016 Nature as indicated on the website?*

**Response:** We have replaced the URL with the citation of Dickinson et al 2016 Nature.

***Comment 5.*** *Page 9, line 151-152: The sentence about W581R and G588S is confusing – consider revising or removing.*

**Response:** As stated above (for the response to comment 2 of Reviewer 2), we agree that the overexpression analysis of previously reported mutants is confusing. Accordingly, we have now removed the data (Fig 1h) and the description (page 9) on previously reported mutants.

***Comment 6.*** *Fig 2c: The authors note that lanosterol was undetectable in the skin of these mice, but in humans, and the tamoxifen-inducible knockout mice, they were able to detect lanosterol and present a ratio. Could the authors speculate on why lanosterol was not detectable? Further, was there any difference in the levels of cholesterol itself?*

**Response:** We supposed that the discrepancy might be based on residual LSS activity. In *Lss^f/f^-K14* mice, *Lss* knockdown occurred in almost all of the cells that expressed the *K14* gene. In contrast, in *Lss^f/f^-K14ERT* mice, the efficiency of *Lss* knockdown would not reach the recombination rate of *Lss^f/f^-K14* mice, as suggested in S11 Fig. [PNAS 1999, PMID 10411913]. Similarly, one of the variants identified in our patients, p.Arg177Gln, seems to be hypomorphic.

We have added experiments to measure cholesterol in the sebum of humans; however, the cholesterol level was low, so we were not able to quantify it. The cholesterol levels in the skin from *Lss^f/f^-K14* mice were detectable, but the levels were not significantly different from what we observed in *Lss^f/f^* mice (S9 Fig).

***Comment 7.*** *Fig 2b, S9 Fig: Can these be presented with individual data points instead, as in other graphs?*

**Response:** We have corrected Figure 2b (including S9 figure in the original manuscript) to clarify the individual data points.

***Comment 8.*** *Fig 2d: Was the decrease in body weight of Lssf/f-K14 mice significantly different from the others? This should be indicated in the text/figure.*

**Response:** Yes, the body weight loss of *Lss^f/f^-K14* mice was significantly different from that of controls. We have added this information to the manuscript (page 10, line195-197) and have modified Figure 2d.

***Comment 9.*** *The figure legends include some results which may be better placed in the main text – please consider revising.*

**Response:** We have addressed this comment in the revised manuscript (page 7 line 132-133) and the figure legends (Figure 1, Figure 4).

***Comment 10.*** *Fig 4c: The authors indicate that lanosterol levels are not significantly different – could the authors comment on why they are unchanged? Are total cholesterol levels changed?*

**Response:** That is an interesting query. Our speculation is that there is supplementation from surrounding tissue or circulation. The *Lss^f/f^-K14ERT* mice are conditional knockouts. Thus, lanosterol could be produced by LSS in tissue in which the keratin 14 promoter does not function. Indeed, as shown in S11 Fig, immunohistochemistry showed that LSS protein was expressed at low levels in the subcutaneous tissue; in this tissue, Cre protein is not supposed to be expressed under the K14 promotor. Further, lanosterol might be delivered to skin via systemic blood flow. We also observed a severe phenotype in parts of the body with few subcutaneous tissues, such as the ears and tail (Fig 3b), consistent with our speculation (see also the discussion section, page 14, line 287-291). We did not observe total cholesterol levels in the sebum of mice because of the limited amount of samples.

***Comment 11.*** *S7 Fig: What is the large peak? Is it cholesterol?*

**Response:** We do not identify the origin of the large peak so far. Because cholesterol was not simultaneously measured in the assay, we are not sure whether the peak is cholesterol.

***Comment 12.*** *S12 Fig: What happens if control mice are treated with a topical application of 4-hydroxytamoxifen?*

**Response:** We also treated littermates (n = 4) with a topical application of 4-hydroxytamoxifen, but they did not show hair loss. This explanation has been added on page 11, line 232-234.

***Comment 13.*** *Some of the supplementary material could be included in the main figures e.g. S5, S7, S9 – I will leave this for the authors to consider.*

**Response:** We appreciate the reviewer’s comment. We have included the supplementary material from S9 Figure in Figure 2b (right).

***Typographical/visual comments:***

***Comment 1.*** *Page 5, line 66: cholesterol and intermediates are “precursors” to vitamin D, etc, rather than “components” of. Please rephrase.*

**Response:** We have replaced “components” with “precursors” in the revised manuscript.

***Comment 2.*** *Page 8, line 135: The mutation is mislabelled as Glu – should be Gln.*

**Response:** We have changed “p.Arg177Glu” to “p.Arg177Gln”.

***Comment 3.*** *Page 13, line 226: Should this be an increased ratio rather than decreased?*

**Response:** We have changed “decreased” to “increased”.

***Comment 4.*** *In some places, LSS is referred to as lanosterol synthase, but in others it is lanosterol synthetase – please be consistent.*

**Response:** We have changed “lanosterol synthetase” to “lanosterol synthase” throughout the text.

***Comment 5.*** *Page 8, line 145: Please define EB-LCLs (and PBMCs page 18, line 342 and fig 1 legend).*

**Response:** We have declared in page 8, line 157 that Epstein-Barr virus-transformed lymphoblastoid cell lines are EBV-LCLs throughout the text.

***Comment 6.*** *Fig 3b: Please label what the top and bottom pictures represent.*

**Response:** As suggested, we have modified Fig 3b and the legend as follows:

“The peak of hair loss in a *Lss^f/f^-K14ERT* mouse was 5 weeks after the tamoxifen treatment (top). The hair had spontaneously regrown 7 weeks after the treatment (bottom).”

***Comment 7.*** *Fig 4a,b,c: For clarity, please consider labelling mouse/lens/H&E/immunohistochemistry.*

**Response:** We have added the labeling in Fig 4.

***Comment 8.*** *S4 Fig: Please indicate which patient.*

**Response:** We have indicated in the legend that the data in S4 Figure are from patient II-3.

***Comment 9.*** *Fig 1f: Please align labels over bands.*

**Response:** We have aligned the labels.

To Senior Publications Assistant

*-Please remove the funding information from your manuscript and instead ensure that this information has been entered correctly into the Financial Disclosure section of the online submission form.*

**Response:** We have confirmed the funding information had been removed from our manuscript. We have also confirmed the information has been entered correctly into the Financial Disclosure section of the online submission form.

*-Please remove your Competing Interests from your manuscript and instead ensure that this information has been entered correctly into the Competing Interests section of the online submission form.*

**Response:** We have removed Competing Interest from our manuscript. We have ensured that this in information has been entered correctly into the Competing Interests section of the online submission form.

*-We notice that you used “data not shown” 4 times in the manuscript, on pages 9 and 10. PLOS Genetics recently updated its data access policy and now requires that all data be either published with the manuscript or made available in a publically accessible database. We ask that you either amend the supplementary material to include the referenced data or remove as many of the references as possible. Please include an explanation of how you have met this request (i.e. whether you have simply removed the instances because they are not integral to the manuscript, or whether you have provided further supporting information files) at the end of your response to reviewers. Please note that we will not be able to pass your manuscript through our technical checks unless this is accompanied by this explanation.*

**Response:** We have amended the four “data not shown” as follows:

Page 9, Line 176: We have provided the data in S3 table.

Page 10, Line 186: We have provided the data in S4 table.

Page 30, Line 661: We have removed the instance because the data is not integral.

Page 34, Line 759: We have removed the instance because the data is not integral.
