# Supplementary material for: Metabolic and pathologic profiles of human LSS deficiency recapitulated in mice
Source: PLoS Genet. 2020 Feb 26;16(2):e1008628. doi: 10.1371/journal.pgen.1008628 (PMC7062289; doi:10.1371/journal.pgen.1008628)
Supplement: Attachment — Submitted filename: Response_to_Reviewers_LSS_PG_20200122_ak_ATSKSJ-6.docx [file pgen.1008628.s023.docx]

Point-by-point answers

**EDITORS**

*Editor comments to author:*

*Thank you for your revised manuscript, which is now acceptable for publication pending the clarification of the textual description of Fig. S13, as suggested by reviewer 2.*

**Response:** Thank you for highlighting the concerns to address in this revision.

**REVIEWER #1**

*The authors have extensively addressed all reviewers' concerns and have added new data to support their findings. Overall, this study is an interesting one; and the data presented here will act as a springboard for further investigations, particularly mechanistic ones, into disorders due to cholesterol biosynthesis defects.*

**Response:** Thank you for your very positive comments.

**REVIEWER #2**

*In the manuscript entitled "Metabolic and pathologic profiles of human LSS deficiency recapitulated in mice ", the authors demonstrate disruption of lanosterol synthetase in an inducible mouse model of LSS deficiency results in biochemical deficits and tissue specific changes present within affected human subjects. The work presented within the manuscript is important to the fields of cholesterol biosynthesis defects, rare disease research, and epidermal function. The authors nicely demonstrate the identification of defined mutations within a small patient cohort of LSS deficiency and recapitulate some of the tissue specific deficits within a newly derived Lss epidermal-specific knockout mouse.

From a positive perspective, the authors attempted to answer the issues raised by reviewers after the initial submission. The manuscript is improved from the first submission and the authors have added some additional supplementary data, attempted to answer biochemical and signaling questions posed by reviewers, and clarified the text. However, as noted in my initial review, I still believe the work as presented is lacking a significant advancement forward for LSS deficiency. Though the study has demonstrated loss of LSS function within the epidermis results in neonatal lethality and lens specific Lss knockout induces tissue phenotypes, there is still a lack of data demonstrating the mechanisms downstream of Lss loss leading to lethality or tissue phenotypes.*

**Response:** We appreciate the reviewer’s comments.

*- Biochemically, there appears to be no loss of epidermal cholesterol (Fig S9) which would mechanistically explain the barrier deficiency presented in Figure 2. It’s possible 2,3-epoxysqualene accumulation at picomolar/mg levels as demonstrated in Figure 2 could exert oxidative stress on the epidermis, inducing apoptosis or lipid peroxidation. However, this is not explored. While I am distinctly aware of the difficulty with these types of analyses in limited tissue samples, these are critical data.*

**Response:** We agree that for further elucidation of the mechanism underlying lanosterol synthase (LSS) deficiency, it is crucial to determine whether substrate accumulation, product deficiency, or both are responsible for epidermal symptoms. We would like to resolve this concern in future studies.

*- While the authors did test LSS effects on SHH and Wnt signaling in MEF isolations, no deficits were identified. It’s not clear if this lack of effect is due to a true maintenance of signaling in the absence of LSS activity or limitations due to the assay. Possible explanations that come to mind include cell type of choice representing mesodermal vs ectodermal lineage of the epidermis, method of LSS deletion via AAV1 (though the qPCR analysis demonstrates fairly efficient knockdown of Lss), or limitations of the qPCR assay used. The methodology suggests MEFs were isolated with FBS, AAV1 transduction performed, and RNA isolated 3 days post-transduction. Was the biochemical defect confirmed at this time point? Further, since both SHH and Wnt signaling utilize morphogen gradients through ligand secretion by one cell type and generally ligand-responsive signaling in a separate cell, I believe IHC analyses specific to the epidermis for SHH and/or Wnt responsiveness would be a better assay of choice. However, based on a lack of cholesterol loss within the epidermis (Fig S9), it's not clear if either of these pathways would be substantially disrupted in this tissue.*

**Response:** Thank you for your insightful comments. Unfortunately, we did not measure the sterol intermediates or cholesterol in mouse epithelial fibroblasts. As you have mentioned and to which we have referred in our previous rebuttal letter, spatial or sequential analysis of the mouse skin, such as immunohistochemistry analysis, may be required to provide evidence of abnormal Wnt/Shh signaling.

*In my view, any of these three outcomes would represent a significant advancement for Lss deficiency:*

*1) identification of causative pathways downstream of LSS loss*

*2) determining the biochemical changes responsible for the pathological findings*

*3) correction (or prevention) of pathological findings.*

*Any of these would be acceptable. But as the work currently stands and in comparison to previously published studies on LSS, the major finding of epidermal or lens-specific loss of Lss with confusing biochemistry (no cholesterol loss) and no well-defined mechanism is troublesome.*

**Response:** We agree that the three outcomes that you have pointed out would be important in elucidating the mechanism underlying LSS deficiency, and we must investigate these issues in future studies. However, we consider that these concerns are beyond the scope of this study, and it would be appreciated if you recognized this.

***Minor issues to clarify related to newly presented data.***

*S13 - “There were fewer retinal ganglion cells in Lssf/f-Pax6 mice than there were in Lssf/f mice. In contrast, the outer nuclear layers were not found to be different between Lssf/f-Pax6 and Lssf/f mice.”*

*” – this is not actually quantified or explained well. What does ‘different’ mean?*

**Response:** We have evaluated the thicknesses of the outer nuclear layers. To clarify our observation, we have changed “In contrast, the outer nuclear layers were not found to be different between *Lss^f/f^-Pax6* and *Lss^f/f^* mice.” to “In contrast, there was no obvious difference in the thickness of the outer nuclear layers between *Lss^f/f^-Pax6* and *Lss^f/f^* mice.” (page 35, line 779–780).

**REVIEWER #3**

*The authors have done their best to address comments by the reviewers. The measurement of additional sterols would indeed be interesting, but is quite difficult and so it is understandable that this has not been as extensive as hoped.*

**Response:** We are grateful for your positive comments and constructive suggestions throughout the review process.
